# Commentary: Impact of resistance training intensity on body composition and nutritional intake among college women with overweight and obesity: a cluster randomized controlled trial

**DOI:** 10.3389/fpubh.2025.1653392

**Published:** 2025-09-29

**Authors:** Joshua L. Keller, Sarah E. Deemer, Gian Luca Di Tanna, Sherri L. Pals, Kevin R. Fontaine, David B. Allison

**Affiliations:** ^1^Applied Physiology Laboratory, Department of Kinesiology, Health Promotion and Recreation, University of North Texas, Denton, TX, United States; ^2^Department of Business Economics, Health and Social Care, University of Applied Sciences and Arts of Southern Switzerland, Manno, Switzerland; ^3^Independent Consultant, Atlanta, GA, United States; ^4^Department of Health Behavior, School of Public Health, University of Alabama at Birmingham, Birmingham, AL, United States; ^5^Children's Nutrition Research Center, Baylor College of Medicine, Houston, TX, United States; ^6^School of Public Health, Indiana University, Bloomington, IN, United States

**Keywords:** statistics, cluster randomized controlled trials, generalized estimating equation, random effects model, experimental design

## Introduction

Cluster randomized controlled trials (cRCTs) are multilevel experimental designs in which groups of participants are randomly assigned to a condition (e.g., an exercise intervention) such that all individuals nested in a naturally occurring group (i.e., cluster) receive the same treatment (1–3). This design is often utilized when individual randomization is not feasible for various reasons (e.g., concerns about contamination of study conditions, logistical difficulties). There are several factors to consider when selecting this design (1, 4, 5); one of the most important is the number of clusters per experimental arm (6, 7). At a minimum, at least two clusters must be randomized to each condition (1), just as more than one participant must be assigned to a condition during traditional individually randomized RCTs. Many investigators experience resource constraints (e.g., funding), leading to fewer clusters. However, resources may be wasted if spent on severely underpowered studies, and regardless of the number of resources, results from cRCTs assigning <2 clusters per condition are uninterpretable as randomized experiments because cluster membership is perfectly confounded with treatment assignment. The optimal number of clusters depends on many factors, including intra-class correlation coefficients, effect sizes, and analysis method (7, 8). Based on our interest in cRCTs (9, 10), we read the Wang et al. investigation (11). We commend this research team for coordinating a 12-week exercise intervention. An investigation of this size requires high effort and dedication. However, we have identified a fundamental flaw in the experimental design, including the statistical approach, which invalidates the corresponding interpretations.

## Experimental and statistical approach considerations

Wang et al. (11) aimed to examine the effects of three different resistance training loads versus a control on body composition and nutritional intake in a sample of women who were overweight or had obesity. The twelve-week resistance programs were designed to be completed using loads defined as the following: (a) Low-intensity (LI): 45–50% one-repetition maximum (1RM), (b) Moderate-intensity (MI): 60–65% 1RM, and (c) High-intensity (HI): 75–80% 1RM. The manuscript (11) defined four campuses in Yichen, Jiangxi, China, which were used to complete this trial, with each campus representing a single cluster: LI was completed at the “new campus” of Early Childhood Teachers College, while MI was completed at the “new campus” and HI was completed at the “old campus” of Yichun College. The control group (CG) was conducted at the Early Childhood Teachers College Gaoan campus. Therefore, the participants were not randomly assigned to a particular campus or group, but instead, each naturally occurring group (i.e., cluster) was assigned a resistance training protocol. To illustrate, Table 1 was created with the data in the manuscript and the data provided in Supplementary material, specifically with the change in percent (%) body fat. We thank the authors for their transparency and for making their raw data publicly accessible. Our intent with Table 1 is to clearly show that one cannot separate whether the differences in mean responses are due to the treatment (e.g., high vs. low loads) or the unique characteristics of the clusters (i.e., campuses) themselves. Said differently, there is no within-cluster variation in treatment assignment; group (treatment) is perfectly collinear with cluster.

**Table 1:** Data depicting the mean change in body fat percentage (%) resulting from 12 weeks of resistance training or control.

**Cluster**	**Condition**	**Mean difference % body fat**	***N***
Campus 1 (new Teacher College)	Low	1.26 (0.622–1.91)	21
Campus 2 (new campus Yichun College)	Medium	2.44 (1.96–2.92)	24
Campus 3 (old campus Yichun College)	High	3.90 (3.21–4.58)	27
Campus 4 (Gaoan campus)	Control	0.974 (0.393–1.55)	30

The parentheses contain the lower and upper bounds of the 95% Wald Confidence Interval for the mean difference (pre-test minus post-test). These data were originally reported in Table 13 (11).

The reported values were generated using a generalized estimating equation (GEE) approach (11) that relies on asymptotic properties, and this can be problematic with a small number of clusters because GEE requires a large sample to calculate accurate standard errors (i.e., sandwich estimator biased downward; underestimates true variability of parameter estimates) (12). Even if a correction is applied to rectify issues with the number of clusters, there is no suitable method to analyze one cluster per condition (13). Thus, the reported values reflect a misapplication of the statistical approach, and their use to justify any conclusions is erroneous (11). Our analysis of the provided data does match the authors' results (11); but, importantly, under these conditions, the analysis does not consider (1) the number of clusters per group, (2) validate whether the design is randomized appropriately, or (3) alert that group-level inference is invalid with a single cluster per condition. Therefore, the results are invalid. Below, we further highlight this.

Consider the following GEE model:


$$\begin{array}{r} Y_{ijt}=\;\mu +{ß}_{1}\cdot \;Time_{t}+{ß}_{2}\cdot Group_{j}+{ß}_{3}\cdot \left(Time_{t}\times Group_{j}\right)\\ +u_{j}+\epsilon_{ijt} \end{array}$$

Where:

- *Y*_*ijt*_: outcome for individual *i* in cluster *j* at time *t*,
- μ: grand mean,
- ß_1_: time effect,
- ß_2_: group (condition) effect,
- ß_3:_ time × group interaction (treatment effect),
- *u*_j_: random (or correlated) cluster effect,
- ϵ_*ijt*_: individual-level error.

In a situation of two campuses and two conditions, if the control is only at the Gaoan campus and the low load is only at the new Teacher College campus, then Group_j_ = Cluster_j_ (i.e., condition = cluster) and the *u*_*j*_ parameter cannot be estimated. Thus, what provokes the change cannot be determined. To correct this, multiple clusters would be needed per condition. We also suggest using a random effects model with an appropriate number of clusters for future cRTC analyses because there are meaningful distinctions between the approaches (i.e., random effects vs. GEE modeling).

## Discussion

The identified fatal flaw of the investigation (11) is that the unit of randomization is the cluster, such that the treatment is being applied at the cluster level, and there is only one cluster per treatment. There is no reported replication of the treatment at the cluster level, no ability to distinguish treatment effects from cluster effects, and zero degrees of freedom for estimating variance between clusters. Varnell et al. (14) have provided a strong warning against one-group-per-condition designs, summarized as “Trials with one cluster per arm should be avoided as they cannot give a valid analysis, as the intervention effect is completely confounded with the cluster effect [(8), p. 2].” Thus, this investigation should be considered an exploratory quasi-experiment, relabeled as such, and no causal inferences should be drawn. An emphasis must also be placed on the fact that any apparent difference between groups needs to be interpreted with substantial caution. Accordingly, per the Committee on Publication Ethics guidelines for handling post-publication critiques (15), a published correction or a retraction and replacement is essential.

## Public health implications

As Wang et al. (11) appropriately described, overweight and obesity continue to contribute substantially to global mortality and the development of chronic diseases, including cardiovascular disease. There is a broad consensus that physical activity, like structured exercise, mitigates the incidence and severity of many adverse health outcomes associated with unhealthy body composition. However, despite its inclusion as a core component of the U.S. Physical Activity Guidelines, the specific benefits of resistance training, particularly in comparison to aerobic training, remain substantially understudied. Evidence from the Aerobic Center Longitudinal Study, which included nearly 12,000 participants at the Cooper Clinic in Dallas, TX, suggested that participation in resistance training was associated with a 20–30% reduction in obesity-related risk (16). These findings highlight the value of conducting randomized controlled trials that investigate resistance exercise as a targeted intervention to combat obesity. Wang et al. (11) sought to contribute to this research agenda by examining how varying resistance training load (e.g., 75–80% 1RM) influenced percent fat and nutritional intake. In addition, and perhaps most importantly, their study focused on female human participants, a population that has been historically underrepresented in biomedical and exercise science research. Collecting data from such groups is essential for developing evidence-based, individualized clinical guidelines for exercise prescription. Although the methodological limitations of Wang et al.'s study preclude its current use in inferences about causal effects, their efforts to investigate this topic are commendable. Their study reflects a growing and necessary interest in understanding how resistance training can be utilized to address obesity, while simultaneously highlighting the need for rigorously designed, randomized controlled trials.
